# Network pharmacology study of Yishen capsules in the treatment of diabetic nephropathy

**DOI:** 10.1371/journal.pone.0273498

**Published:** 2022-09-12

**Authors:** Jingai Fang, Chendan Wang, Jie Zheng, Yuxiang Liu

**Affiliations:** 1 Department of Nephrology, Shanxi Medical University NO.1 Hospital, Taiyuan, China; 2 Department of Nephrology, Shanxi Provincial People’s Hospital, Taiyuan, China; 3 Graduate College, Shanxi Medical University, Taiyuan, China; Bhagwan Mahvir College of Pharmacy, INDIA

## Abstract

**Objective:**

In this study, we used network pharmacology to explore the possible therapeutic mechanism underlying the treatment of diabetic nephropathy with Yishen capsules.

**Methods:**

The active chemical constituents of Yishen capsules were acquired using the Traditional Chinese Medicine Systems Pharmacology platform and the Encyclopedia of Traditional Chinese Medicine. Component target proteins were then searched and screened in the BATMAN database. Target proteins were cross-validated using the Comparative Toxicogenomics Database, and Kyoto Encyclopedia of Genes and Genomes (KEGG) pathway analyses of the target proteins were performed. Then, protein–protein interaction (PPI) analysis was performed using the STRING database. Finally, a pharmacological network was constructed to show the component-target-pathway relationships. Molecular docking was used to analyse the interaction between drug components and target proteins.

**Results:**

In total, 285 active chemical components were found, including 85 intersection targets against DN. In the pharmacological network, 5 key herbs (*A*. *membranaceus*, *A*. *sinensis*, *E*. *ferox*, *A*. *orientale*, and *R*. *rosea*) and their corresponding 12 key components (beta-sitosterol, beta-carotene, stigmasterol, alisol B, mairin, quercetin, caffeic acid, 1-monolinolein, kaempferol, jaranol, formononetin, and calycosin) were screened. Furthermore, the 12 key components were related to 24 target protein nodes (e.g., AGT, AKT1, AKT2, BCL2, NFKB1, and SIRT1) and enriched in 24 pathway nodes (such as the NF-kappa B, AGE-RAGE, toll-like receptor, and relaxin signaling pathways). Molecular docking revealed that hydrogen bond was formed between drug components and target proteins.

**Conclusion:**

In conclusion, the active constituents of Yishen capsules modulate targets or signaling pathways in DN pathogenesis.

## 1. Introduction

Diabetic nephropathy (DN), a common and severe complication of diabetes mellitus (particularly type 2 diabetes mellitus), can eventually develop into end-stage renal disease [1]. Type 2 diabetes mellitus is an underlying cause of kidney failure and tends to lead to hypertension. Estimates show that 6.4% of the global population suffers from diabetes; this percentage is predicted to increase to 7.7% by 2030 [2]. In addition, approximately 20% ~ 40% of all diabetes cases are accompanied by DN [3]. Proteinuria and glomerulosclerosis are the main symptoms of DN [4], and the occurrence and development of DN are linked to metabolic disorders, oxidative stress inflammation, and the pathophysiological mechanisms of fibrosis [5–7]. Clinical treatments to delay the progression of DN focus mainly on controlling blood sugar and pressure, regulating lipid metabolism, anti-oxidation, inhibiting inflammatory reactions, such as using drugs that affect autophagy, etc. [8–10], but these methods do not always cure the condition.

Traditional Chinese medicine has been used to treat DN for several years [11]. Yishen capsules have been widely prescribed and are composed of the *A*. *membranaceus*, *A*. *sinensis*, *E*. *ferox*, *A*. *orientale*, and *R*. *rosea* medicinal herbs. It has been reported that Yishen capsule could reduce proteinuria, protect renal function, and delay progression of early diabetic nephropathy [12, 13]. Additionally, Huangqi-Danggui mixtures have been shown to reduce urinary protein within 24 h, lower urinary albumin, and improve blood glucose [14], and Huangqi capsules have hypoglycemic and antioxidant effects, thus ameliorating DN [15]. Gaoshan-Hongjingtian mixtures may influence the PARP-mediated regulation of NF-κB, thus acting on DN [16]. However, the exact composition and potential pharmacological mechanisms of Yishen capsules remain unclear.

Network pharmacology, an emerging discipline, integrates systems biology and pharmacology to facilitate drug innovation and development and clarify drug treatment mechanisms [17]. In particular, network pharmacology focuses on the complex “drug–gene–target–disease” interactive network [17]. Thus, network pharmacology is a promising approach that can be used to explore the molecular basis of diseases from a multi-dimensional perspective and predict the pharmacological mechanisms of drugs at the molecular and systematic levels. In the present study, the composition of Yishen capsules was analyzed, the effective components were screened, and the component target proteins were identified and cross-validated. Then, Kyoto Encyclopedia of Genes and Genomes (KEGG) pathway analyses of cross-validation targets were conducted. Protein–protein interaction (PPI) analysis was used to identify key target proteins. Finally, pharmacological networks were constructed.

## 2. Materials and methods

### 2.1. Identification of candidate Yishen capsule components

The Traditional Chinese Medicine Systems Pharmacology (TCMSP) database (http://lsp.nwu.edu.cn/browse.php?qc=herbs) was used to acquire data on the chemical components of the five Chinese medicinal herbs found in Yishen capsules, including molecule name, drug half-life, oral bioavailability, molecular mass, and drug likeness. Yishen capsules was composed of *A*. *membranaceus*, *A*. *sinensis*, *E*. *ferox*, *A*. *orientale*, and *R*. *rosea* in a ratio of 3:2:3:2:1 [18]. Then, the Encyclopedia of Traditional Chinese Medicine (ETCM) database (http://www.tcmip.cn/ETCM/index.php/Home/Index/index.html) was used to retrieve small drug molecule information that could not be found in the TCMSP database.

### 2.2. Identification of effective components in Yishen capsules

ADME from the TCMSP database was used to screen Yishen capsules for possible small drug molecules. ADME is the study of the body’s absorption, distribution, metabolism, and excretion process of exogenous compounds. The parameters used included oral bioavailability, drug likeness, and drug half-life. The components were then screened based on the following thresholds: oral bioavailability ≥30% and drug likeness ≥0.18. The ETCM database was used to obtain small molecule information that did not exist in the TCMSP database.

### 2.3. Prediction of Yishen capsule drug targets

After the ADME parameters screening in the last step, the obtained effective components were first converted into PubChem CIDs through the PubChem database (https://pubchem.ncbi.nlm.nih.gov/), and then used as the input items of the Bioinformatics Analysis Tool for Molecular mechANism of Traditional Chinese medicine (BATMAN) (http://bionet.ncpsb.org/batman-tcm/). Default parameters were selected. The target protein gene of each component and the corresponding score were calculated, and the component-target protein relationship pair with score ≥ 5 was screened for further analysis.

### 2.4. Cross-validation of small drug molecule target proteins

The Comparative Toxicogenomics Database (CTD) (updated 2018, http://ctdbase.org/) provides information on the associations between chemicals/genes and diseases to help develop disease mechanisms. The phrase “Diabetic, nephropathies” was used as a keyword based on inference scores to identify genes associated with DN in the CTD database. Then, targets scoring >50 were combined with the targets predicted in the previous step. Following logarithmic transformation, inference scores were acquired to assess the functional relationships among targets in the protein-protein interaction (PPI) network.

### 2.5. Pathway enrichment analysis for DN-related targets of Yishen capsules

Using The Database for Annotation, Visualization and Integrated Discovery online bioinformatics resource (Version 6.8, https://david-d.ncifcrf.gov/) [19], KEGG pathway analysis was conducted on genes that were both target proteins of effective components and disease-related genes. The relevant biological processes were selected with *p* adjusted to ≤0.05 and counts ≥2 being considered significant enrichment results. The most significantly associated top 20 signaling pathways were shown using the bar graph.

### 2.6. Protein–protein interaction (PPI) analysis

Using the Metascape online tool (https://metascape.org/gp/index.html#/main/step1), the PPI network of genes that were both target proteins of effective components and disease-related genes were explored. Then, the BioGrid [20], InWeb_IM [21], and OmniPath [22] interaction databases were used with the following default suggestive values: Min Network Size = 3, Max Network Size = 500. Cytoscape software [23] (Version 3.4.0, http://chianti.ucsd.edu/cytoscape-3.4.0/) was used to construct the PPI network. Further, the Metascape tool performed module mining on the PPI network based on the Molecular Complex Detection (MCODE) algorithm [24]. Finally, after getting PPI modules, KEGG pathway analysis was performed using the ‘clusterProfiler’ (version:3.8.1,http://bioconductor.org/packages/release/bioc/html/clusterProfiler.html) in R package [25]. The Benjamini & Hochberg (BH) method was used to adjust the p values, and *p* < 0.05 was considered the threshold. The most significantly associated top 10 signaling pathways were selected for bubble chart display.

### 2.7. Construction of pharmacological networks and analysis

To further explore the molecular action of Yishen capsules in DN treatment, herbal medicine–component–target proteins–pathway networks were created in Cytoscape. Briefly, drug-effective components, effective components-target protein, and target protein-pathway relationship pairs were input into Cytoscape for network construction. In addition, the module genes identified by the molecular complex detection algorithm in the PPI analysis were selected used to construct a pharmacological network. Nodes of different colors represented the compounds, proteins, or pathways in the pharmacological networks, respectively, and compound–target or target–pathway relationships were presented as edges.

### 2.8. Molecular docking

Four of the target protein nodes in the pharmacological network (AKT1, AKT2, NFKB1, SIRT1) and the small drug molecules beta sitosterol and Stigmasterol targeting them were randomly selected for molecular docking analysis. Information on complexes of target proteins bound to other ligands was downloaded from the Protein Data Bank (PDB) database (http://www.rcsb.org/) [26] and used for subsequent studies. The criterions for screening conformations included the following: (1) Protein structure obtained by x-ray diffraction method; (2) The resolution of the protein structure is less than three; (3) POLYMER ENTITY TYPE is Protein; (4) Ranked first in descending order of score. Then, pymol (Version 2.0 Schrödinger, LLC.) software was used to remove other ligands and water molecules, and the target protein was isolated for subsequent molecular docking. The molecular structure files of small molecules were downloaded from the PubChem Compound database (https://pubchem.ncbi.nlm.nih.gov/) in SDF format and converted to PDB format by pymol for subsequent molecular docking. Then, based on Lamarckian GA algorithm, Autodock software (Version 4.2.6) [27] was used to study the possibility of molecular docking between small drug molecule and targets.

## 3. Results

### 3.1. Composition and component screening

Based on the TCMSP and ETCM databases, the chemical constituents of Yishen capsules were as follows: *A*. *membranaceus* (87), *E*. *ferox* (26), *R*. *rosea* (6), *A*. *sinensis* (126), and *A*. *orientale* (46). Then, after screening the chemical components of the five medicines through a pre-set threshold, 38 of the original 285 chemical components were finally identified as important chemical components and were used for subsequent analysis(Table 1). Specifically, after screening, *A*. *membranaceus*, *E*. *ferox*, *R*. *rosea*, *A*. *sinensis*, and *A*. *orientale* were retained with 20, 2, 6, 2, and 8 effective components, respectively.

**Table 1:** Significant herbal components of Yishen decoction after screening.

Ingredients	Before (number)	After (number)	Source
*A*. *membranaceus*	87	20	TCMSP
*E*. *ferox*	26	2	TCMSP
*R*. *rosea*	6	6	ETCM
*A*. *sinensis*	125	2	TCMSP
*A*. *orientale*	46	8	TCMSP
Total	285	38	

### 3.2. Prediction, screening, and cross-validation of target proteins

**Fig 1:**
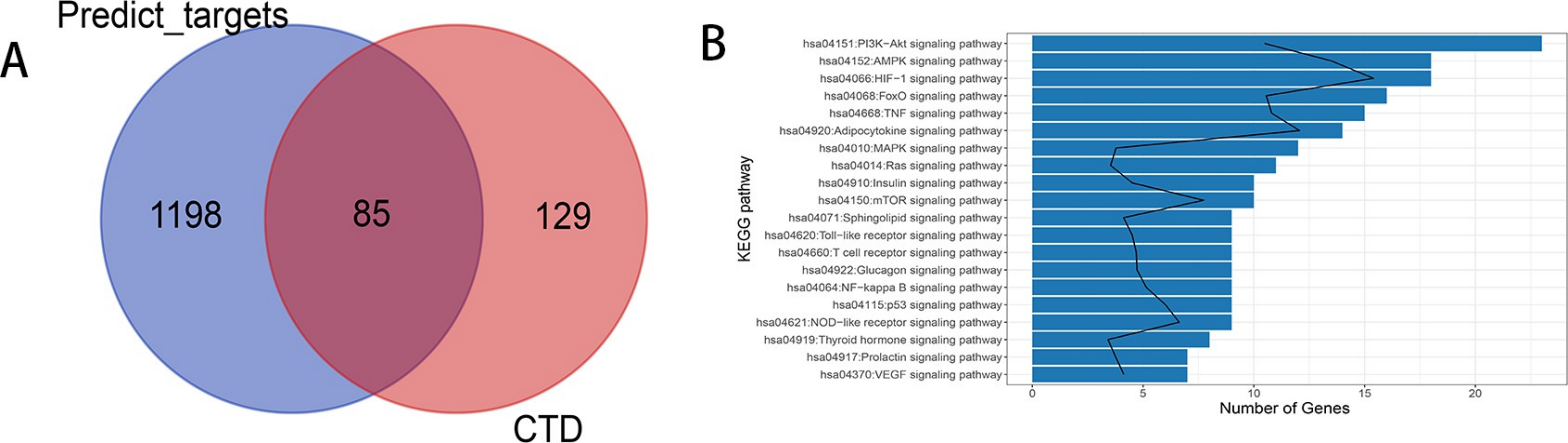
**(A) Venn diagrams of Yishen capsules and cross-validation targets.** Blue represents targets, pink represents genes relevant to DN with inference scores >50 according to the Comparative Toxicogenomics Database, and red represents cross-validation targets **(B) The top 20 pathways of the cross-validation targets.** The bar represents the number of enriched genes, and the black line represents -log10 (p value).

After analysis of BATMAN database, component-target protein pairs were screened according to threshold (scoring ≥5). The relationship pairs included 13 components and 1283 target proteins. Then, these targets were compared with the results for the 214 genes associated with DN with inference scores of more than 50 in the CTD database. Finally, 85 intersected targets of the 1283 targets and 214 genes were obtained (Fig 1A).

### 3.3. KEGG pathway analyses

In total, 102 pathways, including 33 signaling pathways, were identified upon pathway analysis. Moreover, the 20 most significant pathways, such as the hsa04151:PI3K−Akt (23 genes), hsa04152:AMPK (23 genes), hsa04066:HIF−1 (18 genes), hsa04068:FoxO (16 genes), and hsa04668:TNF (15 genes) signaling pathways, are illustrated in Fig 1B. In addition, the hsa04064:NF-kappa B signaling pathway, which contains 9 genes (*VCAM1*, *TNF*, *PTGS2*, *BCL2*, *IL1B*, *NFKB1*, *IKBKB*, *CXCL12*, *CHUK*), was among the top 20 pathways.

### 3.4. PPI analysis

**Fig 2:**
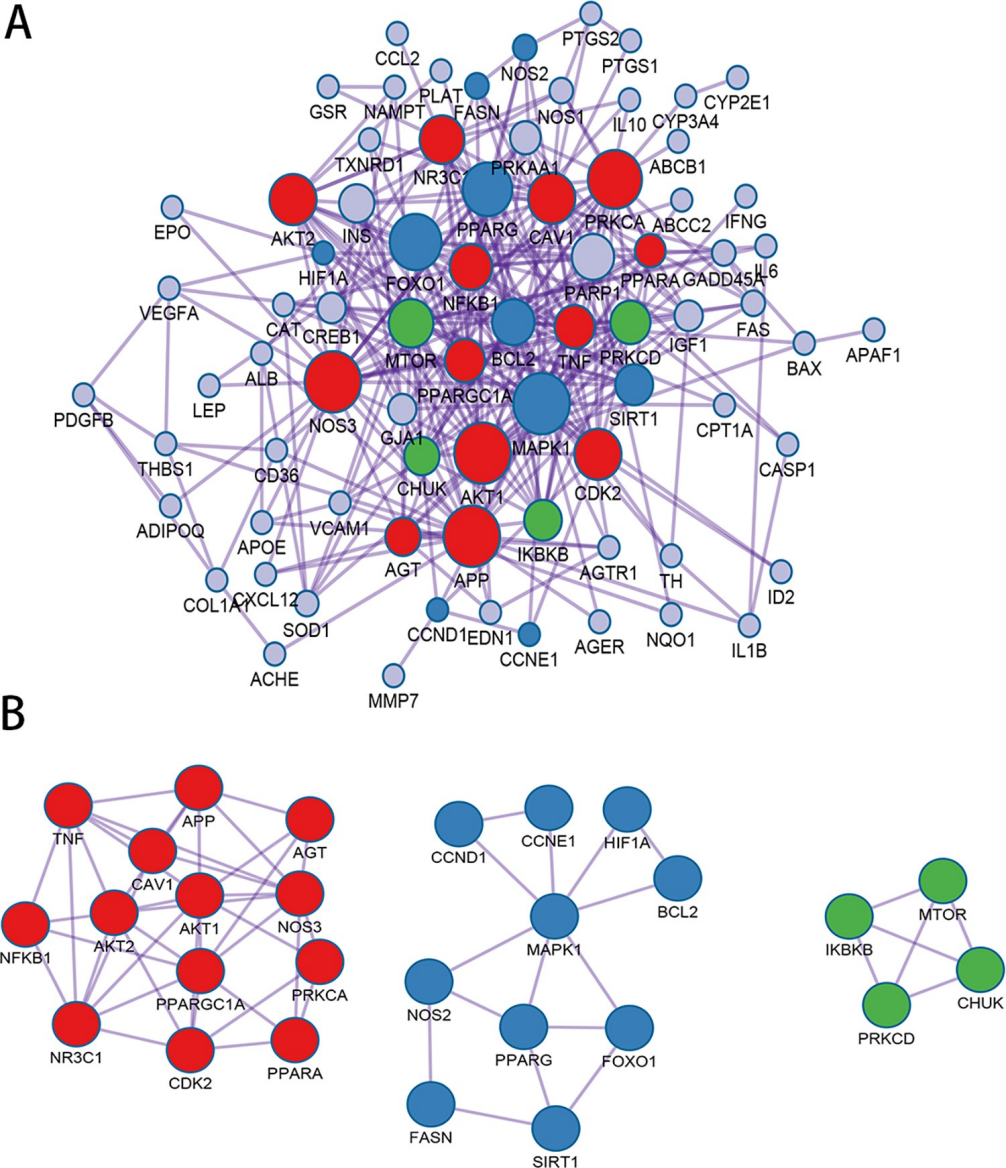
Protein–protein interaction (PPI) network and Molecular Complex Detection (MCODE) components identified related to cross-validation targets. (A) PPI network of proteins encoded by cross-validation targets. (B) Modules selected from the PPI network using MCODE. Red represents MCODE1, blue represents MCODE2, and green represents MCODE3.

**Fig 3:**
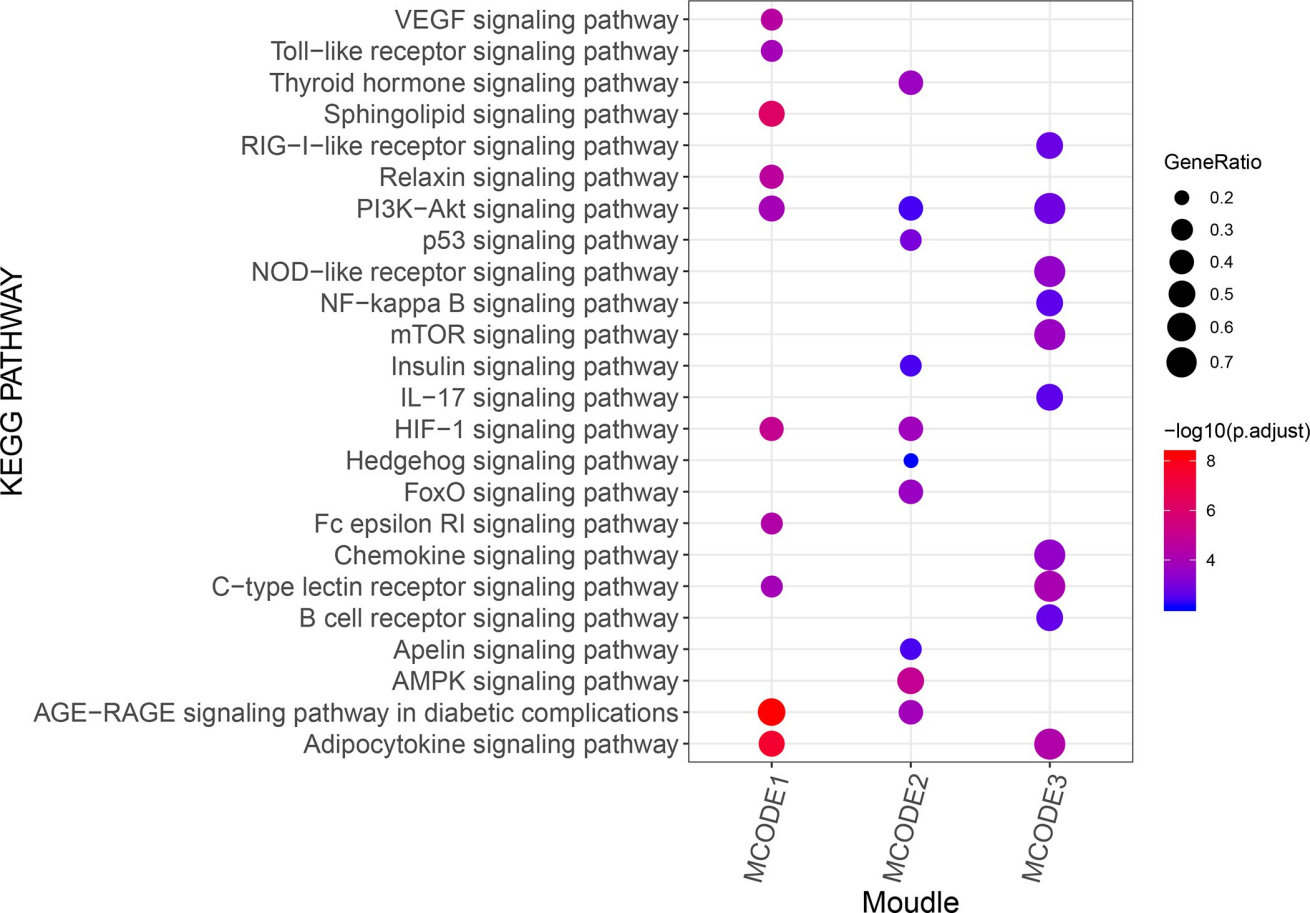
The top 10 signaling pathways in the PPI network. The size of the dot represents the proportion of the number of enriched genes to the total number of genes, with larger dots representing larger proportions; the redder the dot color, the more significant the p value.

As shown in Fig 2A, in total, 77 proteins and 287 edges were identified in the constructed PPI network. MAPK1 (degree = 28), APP (degree = 23), AKT1 (degree = 23), NOS3 (degree = 22), PRKCA (degree = 19), FOXO1 (degree = 18), PPARG (degree = 17), AKT2 (degree = 16), CDK2 (degree = 16), and CAV1 (degree = 16) were identified as potential proteins and were also the top 10 proteins with degree >10. The molecular complex detection algorithm was used to identify the significant modules (Fig 2B), and PARP1 and INS were determined to be the hub proteins. Module 1 contained 13 targets (i.e., APP, AKT1, NOS3, PRKCA, and AKT2), and Module 2 included 10 nodes in addition to MAPK1, FOXO1, PPARG, BCL2, and SIRT1. Moreover, there were four targets in Module 3 (MTOR, PRKCD, IKBKB, and CHUK). In addition, KEGG analysis revealed that 111, 73, and 56 KEGG pathways were enriched in Modules 1, 2, and 3, respectively (Fig 3). Proteins in Modules 1 were mainly enriched in adipocytokine signaling pathway and AGE−RAGE signaling pathway in diabetic complications. Proteins in Modules 2 were mainly involved in AMPK signaling pathway. For the proteins in Modules 3, C−type lectin receptor signaling pathway and adipocytokine signaling pathway were the two important enriched pathways.

### 3.5. Construction of the pharmacological network

**Fig 4:**
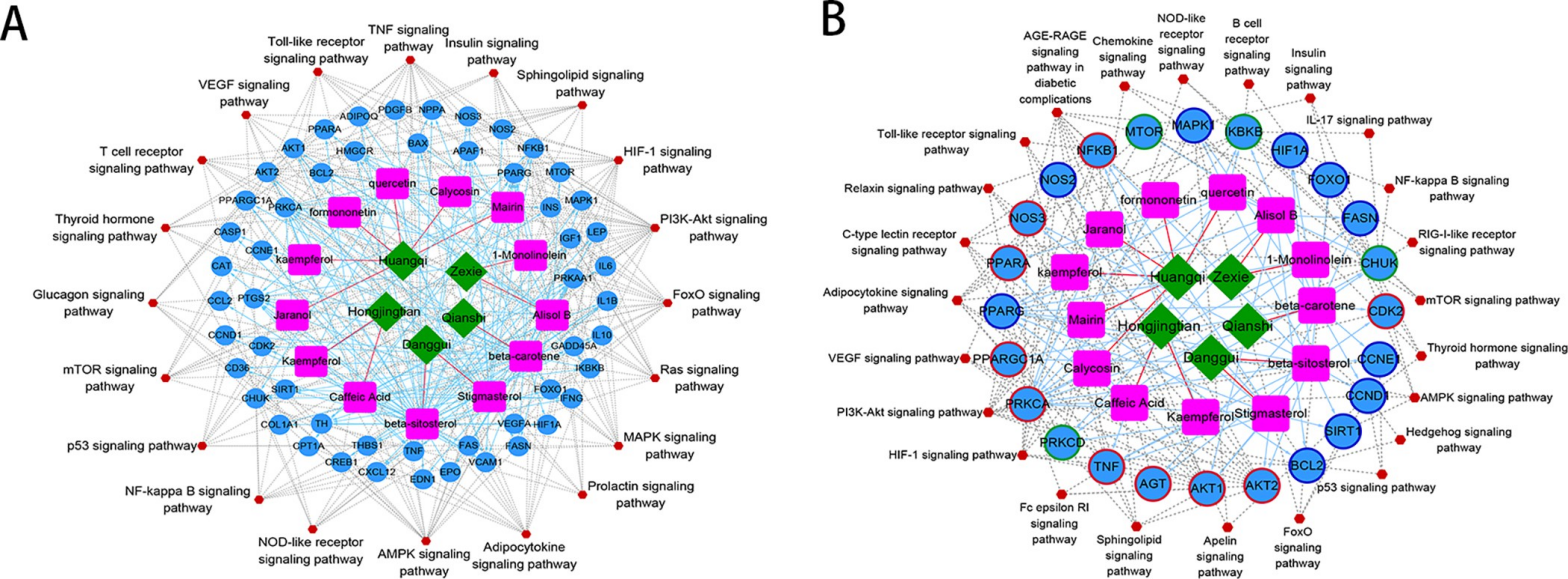
The pharmacological networks for (A) cross-validation targets; (B) cross-validation targets in the protein–protein interaction network. The green rhombus represents herbal medicine; the pink square represents components; the blue dots represent targets; the red hexagon represents the pathways.

The compound–compound target network of the cross-validation targets is shown in Fig 4A, and it includes 91 nodes and 384 edges. There were 5 herbal medicine nodes (*A*. *membranaceus*, *E*. *ferox*, *R*. *rosea*, *A*. *sinensis*, and *A*. *orientale*), 13 chemical component nodes (beta-sitosterol, beta-carotene, stigmasterol, alisol B, mairin, quercetin, caffeic acid, 1-monolinolein, kaempferol, kaempferol, jaranol, formononetin, and calycosin), 53 target protein nodes (such as ADIPOQ, AKT1, AKT2, APAF1, BAX, BCL2, CASP1, CAT, CCL2, and CCND1), and 20 pathway nodes (such as AMPK signaling, adipocytokine, prolactin, MAPK, and Ras signaling pathways) (Fig 4A).

Further, the module genes identified by the molecular complex detection algorithm in the PPI analysis were selected and a pharmacological network containing 65 nodes and 195 edges was constructed. In detail, the network contained 5 herbal medicine nodes (*A*. *membranaceus*, *E*. *ferox*, *R*. *rosea*, *A*. *sinensis*, and *A*. *orientale*), 12 chemical component nodes (beta-sitosterol, beta-carotene, stigmasterol, alisol B, mairin, quercetin, caffeic acid, 1-monolinolein, kaempferol, jaranol, formononetin, and calycosin), 24 target protein nodes (including AGT, AKT1, AKT2, BCL2, NFKB1, and SIRT1), and 24 pathway nodes (including the NF-kappa B, AGE-RAGE, toll-like receptor, and relaxin signaling pathways) (Fig 4B).

### 3.6. Molecular docking of target protein and compounds

**Fig 5:**
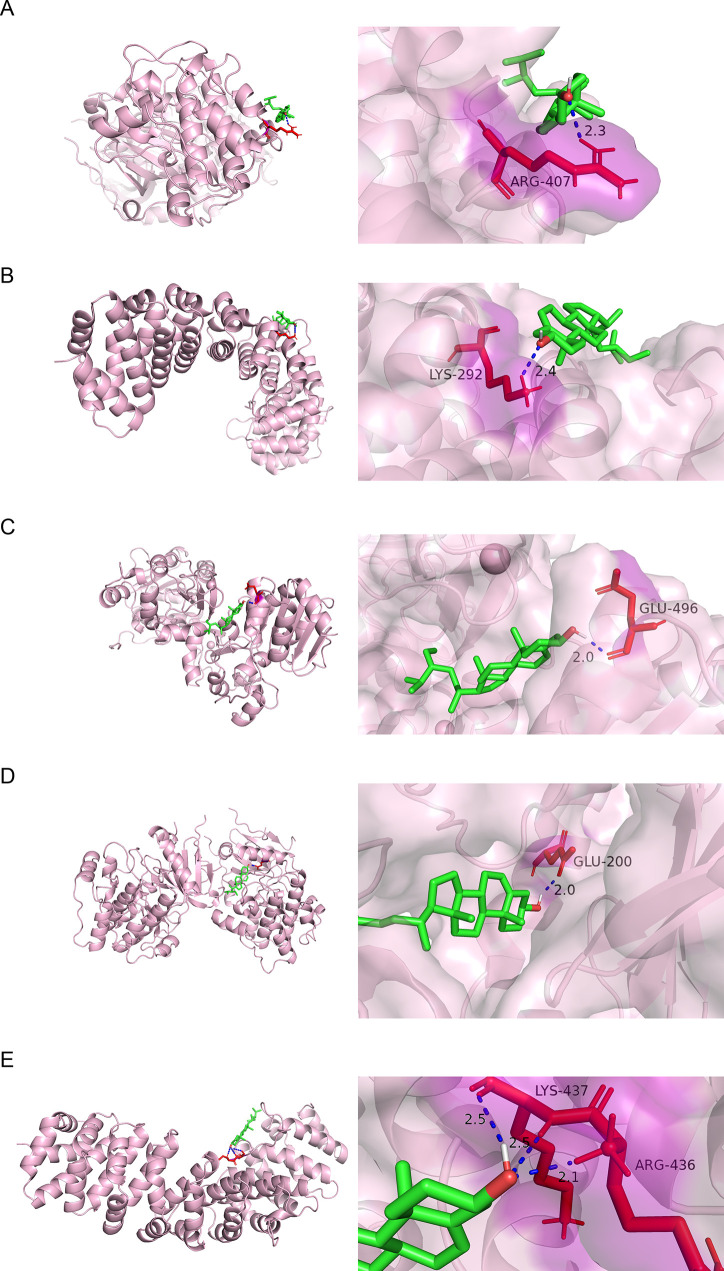
Molecular docking results. (A) Docking results of beta sitosterol targeting AKT2; (B) Docking results of beta sitosterol targeting NFKB1; (C) Docking results of beta sitosterol targeting SIRT1; (D) Docking results of stigmasterol targeting AKT2; (E) Docking results of stigmasterol targeting NFKB1. Light pink represents protein receptors. Green represents small molecule ligands of compounds. Blue line represents hydrogen bonds between small molecule ligand and protein. Dark purple represents amino acid residues that have hydrogen bonds with small molecules of compounds.

The results of molecular docking showed that, in beta sitosterol, hydrogen bond was formed between the ligand and ARG-407 of AKT2 and LYS-292 of NFKB1 (Fig 5A and 5B). There was a hydrogen bond between the ligand of beta sitosterol and GLU-496 of SIRT1 (Fig 5C). In addition, hydrogen bond was formed between the ligand of stigmasterol and GLU-200 of AKT2 (Fig 5D). Fig 5E showed that hydrogen bonds were formed between the ligand of stigmasterol and LYS-437 and ARG-436 of NFKB1. (Results for target proteins and compounds that do not form hydrogen bonds were not shown.)

## 4. Discussion

Diabetic nephropathy increases morbidity and mortality in both type 1 and type 2 diabetes mellitus [28] and is the second most-common cause of chronic kidney disease after chronic glomerular disease [29]. Clinically, microalbuminuria is used as an important index to evaluate DN progression [30]. Hyperglycemia, increased blood pressure, and genetic predisposition are all well-known risk factors of DN [31]. In the present study, following network pharmacology analyses based on PPI targets, a total of 5 key herbs (*A*. *membranaceus*, *A*. *sinensis*, *E*. *ferox*, *A*. *orientale*, and *R*. *rosea*) and 12 key components (beta-sitosterol, beta-carotene, stigmasterol, alisol B, mairin, quercetin, caffeic acid, 1-monolinolein, kaempferol, jaranol, formononetin, and calycosin) were identified in Yishen capsules. These 12 key components were associated with 24 target protein nodes (e.g., AGT, AKT1, AKT2, BCL2, NFKB1, and SIRT1) and 24 pathways.

Network pharmacology analyses indicated that beta-sitosterol, beta-carotene, stigmasterol, alisol B, mairin, quercetin, caffeic acid, 1-monolinolein, kaempferol, jaranol, formononetin, and calycosin play key roles in the progression of DN. Clinical and experimental studies have reported β-sitosterol (24-ethyl cholestene-3-ol) as a naturally occurring plant sterol that possesses antihyperlipidemic and antihyperglycemic properties [32, 33]. Further, Saravanan et al. concluded that the role of β-sitosterol in antidiabetic activity was mainly the result of antioxidant enzymes in the liver [34]. He et al. indicated that kaempferol, stigmasterol, and beta-sitosterol constituted the central node of the compound–compound target network of Liu Wei Di Huang pills in the treatment of type 2 diabetes mellitus [35]. Meanwhile, prior evidence has shown that quercetin inhibits oxidative damage in various tissues of streptozotocin-induced diabetic rats [36, 37]. Oxidative stress has been suggested to be the pathophysiological mechanism underlying DN progression, and quercetin may have antioxidant properties that attenuate DN [38]. Furthermore, quercetin has been shown to reduce the renal fibrosis induced by diabetes [39]. In contrast, a caffeic acid derivative was utilized to protect against renal damage [40] and ameliorate DN by regulating the autophagy pathway in high-fat diet/streptozotocin-induced diabetic rats [41]. Therefore, beta-sitosterol, stigmasterol, quercetin, caffeic acid, and kaempferol were the relevant components of Yishen capsules for the treatment of DN.

In the pharmacological network, AGT, AKT1, AKT2, BCL2, SIRT1, and NFKB1 were important target protein nodes. The production of AGT is involved in DN progression [42]. The T235 AGT polymorphism has been shown to be associated with DN [43]. In addition, a T allele polymorphism in AGT is a genetic risk factor for DN [44]. AKT1 in the renal tubular epithelium and p-Akt1 (Ser(473)) are more prevalent in diabetic patients [45]. AKT2 silencing prevents renal protection in mice with streptozotocin-induced diabetes [46]. Diabetic patients with poor glycemic control exhibit the downregulation of BCL2, which activates the NF-kB pathway, thus leading to the development of nephropathy [47]. The regulation of beclin1/UVRAG/BCL2 could be involved in the cell apoptosis and cell autophagy observed in DN [48]. Therefore, AGT, AKT1, AKT2, and BCL2 may be crucial proteins in the action of Yishen capsules against DN.

SIRT 1 regulates the Bax and Bcl-2 apoptotic proteins in DN [49], and a NFKB1 gene polymorphism (rs28362491) is associated with DN [50]. NFKB1 variations contribute to the development of type 2 diabetes mellitus [51], and the expression of NFκB is upregulated in diabetic patients [52]. SIRT1 and NFKB were both present in the pharmacological network constructed in the present study. Additionally, the NF-kappa B signaling pathway was found to play a key role in DN in the pharmacological network. Previous studies have indicated that the NFκB signaling pathways are involved in the development mechanisms of DN [53, 54]. Our previous study confirmed that Yishen capsule promotes podocyte autophagy through regulating SIRT1/NF-κB signaling pathway to improve diabetic nephropathy [18].

Moreover, in total, 20 pathway nodes were found to be associated with DN in the target network of cross-validation targets. The AMPK signaling pathway improves DN by reducing uric acid, serum albumin, creatinine, and kidney damage [55]. The AMPK signaling pathway has been shown to alter fatty acid oxidation and glucose in C57BL/6 mice with type 2 diabetes [56]. The p38 MAPK signaling pathway plays an important role in modulating cell differentiation, growth, and death [57]. Elevated mRNAs in the PKC-MAPK pathway are essential in the glomerular lesion damage observed in DN [58]. Moreover, the Ras signaling pathway has been demonstrated to affect streptozotocin/nicotinamide mice [59] or diabetes-induced VEGF-mediated nephropathy [60]. Thus, the AMPK and Ras signaling pathways influence the development of DN.

## 5. Conclusion

In conclusion, beta-sitosterol, stigmasterol, quercetin, caffeic acid, and kaempferol were identified as key components of Yishen capsules in the treatment of DN. AGT, AKT1, AKT2, and BCL2 may be important target proteins in the pharmacological network. Moreover, SIRT1 and NFKB1 may interact to regulate DN via the NF-kappa B signaling pathway. Furthermore, the AMPK and Ras signaling pathways are highly important in DN. However, more research is required to further validate these results.
